# Evolution and applications of genome-scale metabolic models in yeast systems biology studies

**DOI:** 10.1093/femsyr/foaf045

**Published:** 2025-08-28

**Authors:** Xiaodan He, Hongzhong Lu

**Affiliations:** State Key Laboratory of Microbial Metabolism, School of Life Science and Biotechnology, Shanghai Jiao Tong University, Shanghai 200240, P. R. China; State Key Laboratory of Microbial Metabolism, School of Life Science and Biotechnology, Shanghai Jiao Tong University, Shanghai 200240, P. R. China

**Keywords:** yeast, genome-scale metabolic models, metabolic engineering, big data analysis

## Abstract

Genome-scale metabolic models (GEMs) can be used to simulate the metabolic network of an organism in a systematic and holistic way. Different yeast species, including *Saccharomyces cerevisiae*, have emerged as powerful cell factories for bioproduction. Recently, with the dedicated efforts from the scientific community, significant progress has been made in the development of yeast GEMs. Numerous versions of yeast GEMs and the derived multiscale models have been released, facilitating integrative omics analysis and rational strain design for different types of yeast cell factories. These advancements reflected the evolution and maturation of yeast GEMs together with a model ecosystem around them. This review will summarize the development and expansion of yeast GEMs and discuss their applications in yeast systems biology studies. It is anticipated that yeast GEMs will continue to play an increasingly important role in pioneering yeast physiological and metabolic studies in coming years.

## Teaser

This review introduces recent advances and applications of yeast genome-scale metabolic models, focusing on the developments since Yeast8 and their applications in systems biology and metabolic engineering.

## Introduction

Systems biology is a discipline dedicated to the modeling and analysis of complex biological systems (Kirschner 2005, Nielsen and Petranovic 2025). Its primary task is to investigate the interaction networks of molecules within organisms and their influence on phenotypes (Hatzimanikatis et al. 2004, Price et al. 2004, Aderem 2005). Unlike traditional reductionist methods that examine biological components in isolation, systems biology seeks to illustrate biological phenomena through the net interactions of all cellular and biochemical components within a cell or an organism. Consequently, accurate representation of the interaction networks of biomolecules is essential to enable the computation of mathematical models (Liu 2005) and to simulate biological behavior (Dobson et al. 2010).

Genome-scale metabolic models (GEMs) are computational frameworks that integrate gene–protein-reaction (GPR) associations for nearly all metabolic genes within an organism, which have the potential to incorporate comprehensive data on stoichiometry, compartmentalization, biomass composition, thermodynamics, and gene regulation (Edwards et al. 2001, O'Brien et al. 2015, Gu et al. 2019). By imposing systemic constraints on the entire metabolic network, GEMs enable researchers to predict cellular responses under diverse conditions (Orth et al. 2010, Heavner and Price 2015). The consistent development of yeast GEMs along with the gradually added strain-specific knowledge has significantly advanced the understanding of yeast metabolic activities, facilitating their applications in fields including metabolic engineering and synthetic biology (Price et al. 2004, Feist et al. 2009, Feist and Palsson 2010, Thiele and Palsson 2010, Lopes and Rocha 2017).

Recent advances in omics profiling technologies have significantly promoted the development of GEMs for yeast due to the accumulation of high-quality datasets. These datasets facilitate the integration of diverse omics data, including transcriptomics, proteomics, and metabolomics information, beyond traditional genomic data. Such integration has led to the creation of multiscale metabolic models, such as enzyme-constrained GEMs (ecGEMs), which incorporate proteomic data to enhance predictive capabilities (Chen et al. 2024b). Furthermore, progress in single-cell transcriptomics for yeast has enabled comprehensive measurements of transcriptome abundances at the single-cell level, providing a robust foundation for reconstructing context-specific models under various physiological conditions, making it possible to develop strain-specific GEMs (ssGEMs) (Zhang et al. 2024). The continuous increase in the availability of rich and high-precision omics data is expected to further improve the accuracy and predictive capability of GEMs, thereby advancing their utility in systems biology and metabolic engineering.

This review will summarize the development of yeast GEMs since the release of Yeast8, and then provide an overview of recent advances in yeast multiscale models. Subsequently, the applications of GEMs in yeast systems biology and metabolic engineering will be introduced, describing how these models support systems biology and metabolic engineering through omics integration, dynamic analysis, and rational strain design. Throughout the review, we will discuss how the development of various advanced models based on GEMs could contribute to a deeper understanding of yeast metabolism in a holistic way.

## Recent development in yeast GEMs since the release of Yeast8

**Figure 1.**
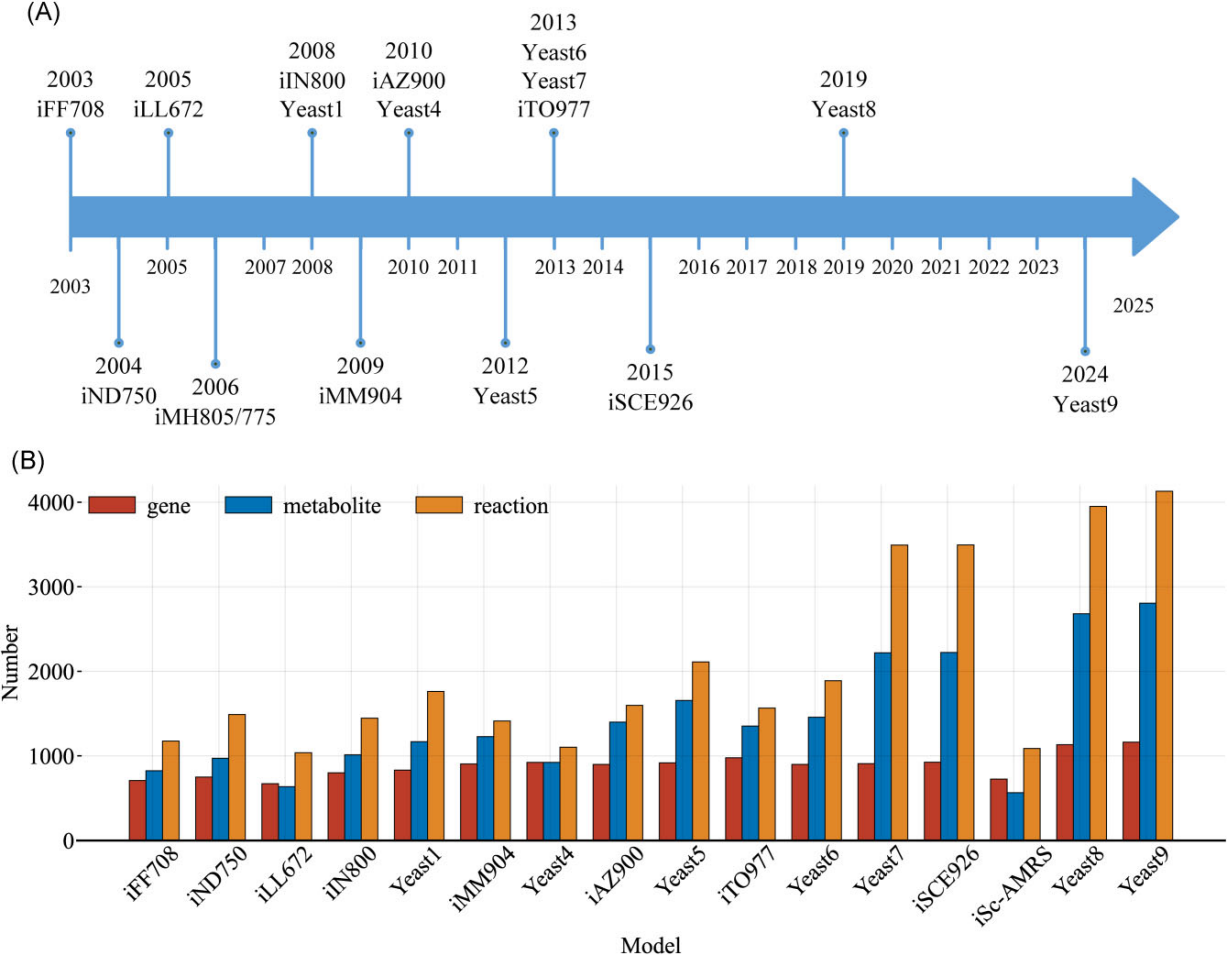
Historical development of yeast GEMs. Since 2003, over a dozen GEMs for *S. cerevisiae* have been published, ranging from iFF708 to Yeast9 (A). The construction and curation of yeast GEMs have been ongoing and progressive. Both the number of genes and the count of metabolites and reactions in these models generally exhibit an increasing trend (B).

The development of GEMs for *Saccharomyces cerevisiae* began in 2003 with the publication of the first model, iFF708, by Förster et al. (2003). Following this, various research groups released multiple versions of metabolic models for *S. cerevisiae* (Duarte et al. 2004, Kuepfer et al. 2005, Herrgård et al. 2006, Nookaew et al. 2008). However, inconsistencies in construction methodologies and data formats across these models hindered data integration, prompting the construction of a consensus model by Herrgård et al. (2008). This consensus model, known as Yeast1, has since undergone continuous updates, with subsequent versions reflecting advancements in our understanding of yeast metabolism. The evolution of these models up to recent iterations has been comprehensively reviewed by Chen et al. (2022) and Lu et al. (2022b) (Fig. 1). These efforts in model creation and curation laid a solid foundation for the maturation of yeast GEMs and promoted their wider applications in fields of systems biology and synthetic biology.

Building on the groundwork established by Yeast1 and its successors, Yeast8, a GEM for *S. cerevisiae* was published in 2019, incorporating enhancements over Yeast7, such as the addition of SLIME reactions and updated GPR associations (Lu et al. 2019). Continuing this trajectory of improvement, the latest consensus model, Yeast9, was released in 2024 (Zhang et al. 2024). Compared with Yeast8, some new updates were merged into Yeast9, such as corrections to mass and charge balances, refined gene associations, and the inclusion of thermodynamic parameters, enabling more accurate and comprehensive simulations of yeast metabolism. Yeast9 serves as a powerful tool for yeast systems biology research. For example, by integrating single-cell transcriptomics, Yeast9 could be used to analyze the mechanisms of how yeast adapts to high osmotic pressure. These advancements illustrate how ongoing refinements in yeast GEMs continue to expand their utility and precision.

**Figure 2.**
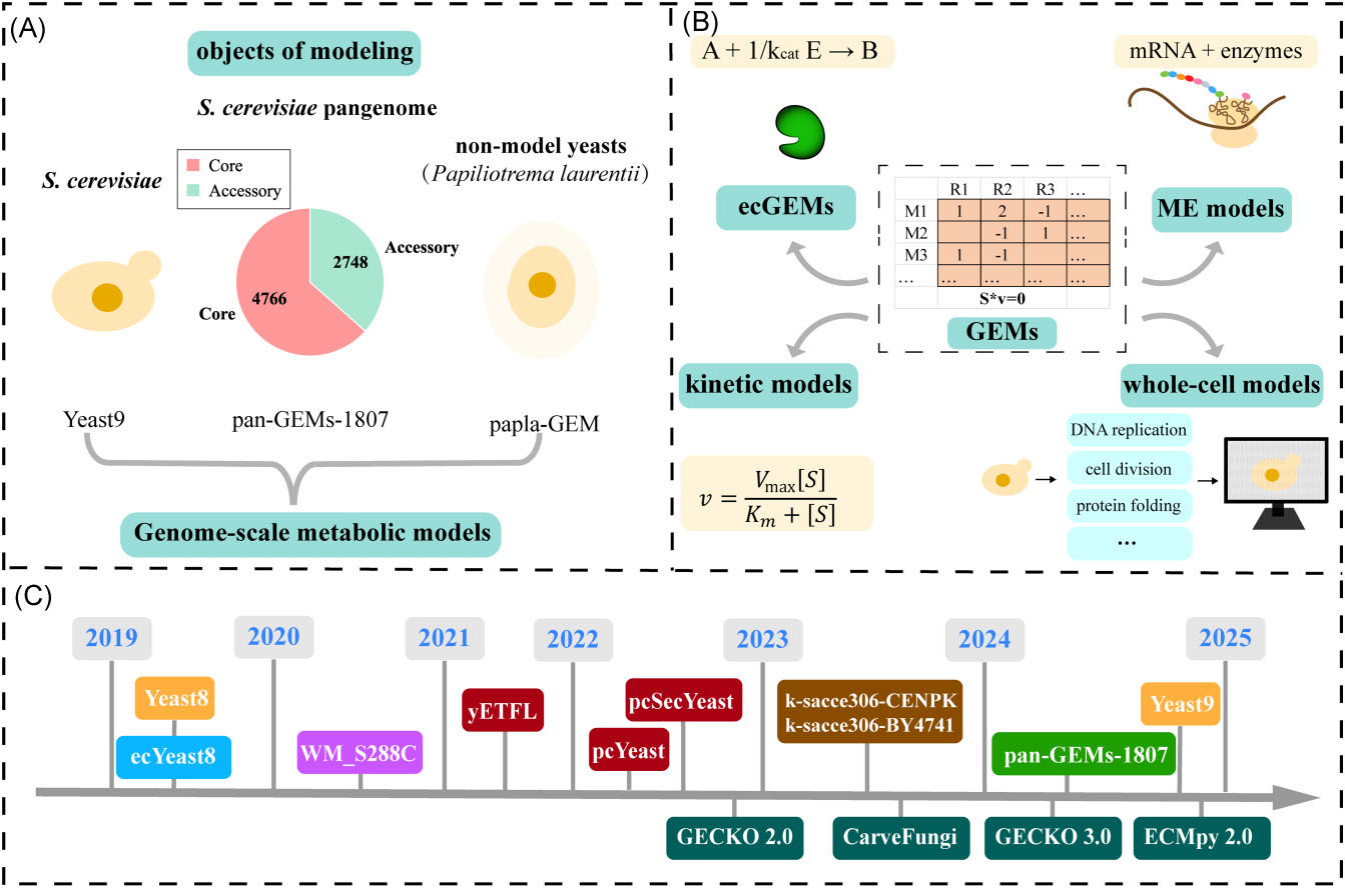
Evolution of yeast GEMs from single-strain to multistrain, as well as from single-scale to multiscale. ssGEMs and pan GEMs developed based on the yeast-GEMs (e.g. Yeast9) (A). Building on GEMs, various multiscale metabolic models could be developed. These include ecGEMs that incorporate enzyme quantities and kinetic constraints, ME models that consider protein translation processes, kinetic models that include Michaelis–Menten equations and other kinetic expressions, and whole-cell models that encompass cellular physiological processes such as DNA replication, cell division, and protein folding (B). Timeline of key milestones in yeast GEMs development from 2019 to 2024, including classical GEMs and multiscale GEMs (C).

The classical GEMs are constructed mainly based on the reference genomes of *S. cerevisiae* S288c, which cannot account for the genetic diversity from different strains. Thus, it is essential to develop pan models using pangenomes that incorporate accessory genes missing from reference genomes, such as panYeast8 (Lu et al. 2019) and pan-GEMs-1807 (Wang et al. 2025) (Fig. 2A). As one typical example, Wang et al. (2025) developed pan-GEMs-1807 based on the pan-genome of 1 807 *S. cerevisiae* isolates, which could cover ∼98% of the genes in *S. cerevisiae* CEN.PK. Then, combined with a gene presence matrix, pan-GEMs-1807 was used to generate 1807 ssGEMs by removing the absent genes, as well as reactions associated with those absent genes. These ssGEMs demonstrate high quality, with 85% successfully simulating growth under glucose minimal media conditions. Furthermore, these ssGEMs revealed significant metabolic differences among strains from distinct niches, providing valuable insights into how *S. cerevisiae* adapts through genomic and metabolic reprogramming.

Except for *S. cerevisiae*, nonmodel yeasts such as *Pichia pastoris, Yarrowia lipolytica*, and *Starmerella bombicola* are also pivotal in industrial biotechnology. Thus, GEMs have been built for those valuable yeast species. For example, iPN730 was built for *Lachancea kluyveri* (Nanda et al. 2020), *iIsor*850 was built for *Issatchenkia orientalis* SD108 (Suthers et al. 2020), papla-GEM was built for *Papiliotrema laurentii* (Ventorim et al. 2022), and iEM759 was built for *S. bombicola* (Motamedian et al. 2024). The application of these models could be exemplified by the iEM759 model, which, optimized for high-sugar conditions, enables the evaluation of strategies in coupling sophorolipid production with cellular growth (Motamedian et al. 2024). Furthermore, a comprehensive overview of recent progress in nonmodel yeasts metabolic modeling could be found in a recent review by de Almeida et al. (2024).

Notably, the reconstruction of GEMs requires initially mapping genome sequences to a knowledge database or high-quality GEMs of closely related species to generate a draft model encompassing all identified metabolic reactions. Subsequently, the draft model is refined to produce a high-quality model. In addition to manual construction of GEMs and derivation from pan-genome models, some existing computational tools, such as RAVEN (Wang et al. 2018) and CarveFungi (Castillo et al. 2023), have facilitated the automated reconstruction of draft GEMs for any genome-sequenced yeast species by leveraging information such as genome annotations. For instance, the reconstruction of the previously mentioned papla-GEM model utilized the RAVEN toolbox (Ventorim et al. 2022). Moreover, Lu et al. (2022a) employed RAVEN v2.0 to construct draft GEMs for 332 yeast species using genomic and proteomic data. Although these models could not directly predict cellular phenotypes based on genetic or physiological constraints, they provided insights into the evolutionary drivers of trait diversity. Similarly, Pettersen et al. (2023) used CarveFungi to build GEMs for five nonmodel yeasts to probe fermentative capabilities. In summary, these automatically constructed models are expected to serve as good starting templates to build high-quality models for nonmodel yeast species through rounds of manual curation.

## Advances in multiscale yeast metabolic models

Classical GEMs face challenges in accurately simulating cellular metabolism due to limited constraints. To address these limitations, various kinds of multiscale models have been developed to narrow the solution space of metabolic models, thus overcoming the drawbacks of classical GEMs (Fig. 2B and C). These models incorporate additional constraints, such as enzymatic or kinetic constraints, or integrate omics data, such as proteomics and transcriptomics, building upon traditional GEMs (Bi et al. 2022) (Table 1).

**Table 1.** Characteristics of multiscale yeast metabolic models.

Model type	Characteristics	Representative model	References
ecGEMs	Models incorporating enzyme kinetic parameters and enzyme abundance as constraints	ecYeast8	Lu et al. (2019)
ME models	Models integrating metabolic and gene expression pathways	yETFL, pcYeast, pcSecYeast	Li et al. (2022a), Oftadeh et al. (2021), and Elsemman et al. (2022)
)Kinetic models	Models incorporating dynamic changes in reaction rates	k-sacce306-CENPK, k-sacce306-BY4741	Hu et al. (2023)
Whole-cell models	Models simulating the behavior of an entire living cell by capturing complex interactions among proteins, metabolites, genes, and regulatory networks	WM_S288C	Ye et al. (2020)

ecGEMs are GEMs that extend traditional GEMs by setting the enzyme kinetic parameter and enzyme abundance as additional constraints. For instance, a method that enhances a GEM with enzymatic constraints using kinetic and omics data (GECKO) can be used for converting GEMs into ecGEMs (Chen et al. 2024b, Sánchez et al. 2017, Domenzain et al. 2022). GECKO integrates enzyme molecular weight, abundance, and turnover numbers (*k*_cat_) into metabolic models by including enzymes as components of reactions and introducing pseudometabolites and pseudoreactions to constrain total protein allocation. Compared to conventional GEMs, ecGEMs exhibit superior simulation performance. For example, when predicting growth rates across various carbon and nitrogen sources, Yeast8 exhibits a mean error of 120% relative to experimentally measured maximum specific growth rates, whereas ecYeast8, which was derived from Yeast8 using the GECKO framework, achieves an average error of 42% (Lu et al. 2019). In addition to GECKO, another workflow for constructing an enzymatic constrained metabolic network model (ECMpy) is another tool for constructing ecGEMs, distinguished by its approach to imposing total protein constraints without altering the stoichiometric matrix (Mao et al. 2022, Mao et al. 2024).

While ecGEMs account for the constraints from enzyme kinetics and abundances, they do not account for the costs of protein synthesis. In contrast, metabolism and expression models (ME models) incorporate stoichiometric representations of transcription and translation, integrating metabolic and gene expression pathways to compute coupled proteome allocation and metabolic phenotypes (O'Brien et al. 2013). For example, using the expression and thermodynamics-enabled flux (ETFL) approach (Salvy et al. 2020), Oftadeh et al. (2021) developed an ME model for yeast, named yETFL. Building upon the Yeast8 model, yETFL incorporates constraints related to metabolism, thermodynamics, catalysis, expression, and allocation, integrating reaction fluxes, enzyme efficiencies, transcription, translation capacities, and macromolecular content into a unified steady-state framework. Compared to ecGEMs generated by GECKO (Sánchez et al. 2017), yETFL could capture the Crabtree effect solely by integrating experimentally measured data, without imposing additional parameter restrictions on enzyme availability. In a complementary approach, Elsemman et al. (2022) introduced a comprehensive proteome-constrained yeast model (pcYeast) that enhances the prediction performances by constraining compartment-specific protein pools. This model reveals that mitochondrial constraints drive the Crabtree effect under glucose limitation, while cytosolic volume constraints dictate overflow metabolism under sugar excess, showcasing advanced multiscale models help to probe the mechanisms underlying metabolic adaptation in eukaryotic cells. Similarly, Li et al. (2022a) constructed a proteome-constrained genome-scale protein secretory model of yeast *S. cerevisiae* (pcSecYeast) based on pcYeast encompassing the detained steps in protein synthesis and secretion. This model integrates metabolic and protein maturation processes, from cytoplasmic nascent peptides to mature forms in target compartments, using coupled constraints linking energy and substrate availability to protein synthesis demands. It also accounts for competition between recombinant and native proteins in secretion pathways.

While most models mentioned before rely on steady-state assumptions, which limit the simulation of time-dependent behaviors, kinetic models address this limitation by incorporating dynamic changes in reaction rates, thereby capturing transient responses and regulatory mechanisms (Saa and Nielsen 2017). In 2023, Mishra et al. (2023) developed a kinetic model for lipid metabolism in *S. cerevisiae*, which includes fatty acid biosynthesis, glycerophospholipid metabolism, ceramide metabolism, storage lipid formation, and sterol ester synthesis, as well as the production and transport of target chemicals such as fatty acids and fatty alcohols. The model has been applied to design mutant strains that overproduce fatty alcohols and to interpret both successful and unsuccessful strain designs reported previously. Additionally, Hu et al. (2023) advanced kinetic modeling in *S. cerevisiae* by constructing two strain-specific models, k-sacce306-CENPK and k-sacce306-BY4741, based on Yeast8. These models could capture metabolic differences between strains including glucose and oxygen uptake rates, ethanol production, and ergosterol content, which were validated through single-gene deletion simulations that revealed strain-specific flux distributions. The yeast kinetic models show advantages in depicting cellular dynamic metabolic rewiring, thus contributing to the design of optimized strains for industrial applications.

In addition to the models mentioned above, whole-cell models are computational frameworks designed to simulate the behavior of an entire living cell by capturing the complex interactions among various cellular components, encompassing proteins, metabolites, genes, and regulatory networks. These models aim to offer a comprehensive modeling of cellular processes at the systems level, which could help to explore how distinct molecular entities collaboratively perform functions such as metabolism, signal transduction, and gene expression (Tummler and Klipp 2024). As the first example, Ye et al. (2020) built the WM_S288C whole-cell model on iTO977 for *S. cerevisiae*, integrating 15 cellular states and 26 processes, such as metabolism, DNA replication, protein folding, and cell division. WM_S288C simulations revealed how nonessential genes regulate nucleotide levels to control cell growth, which provides chances to elucidate genotype-phenotype relationships at a larger level.

## Applications of yeast genome-scale metabolic models

**Figure 3.**
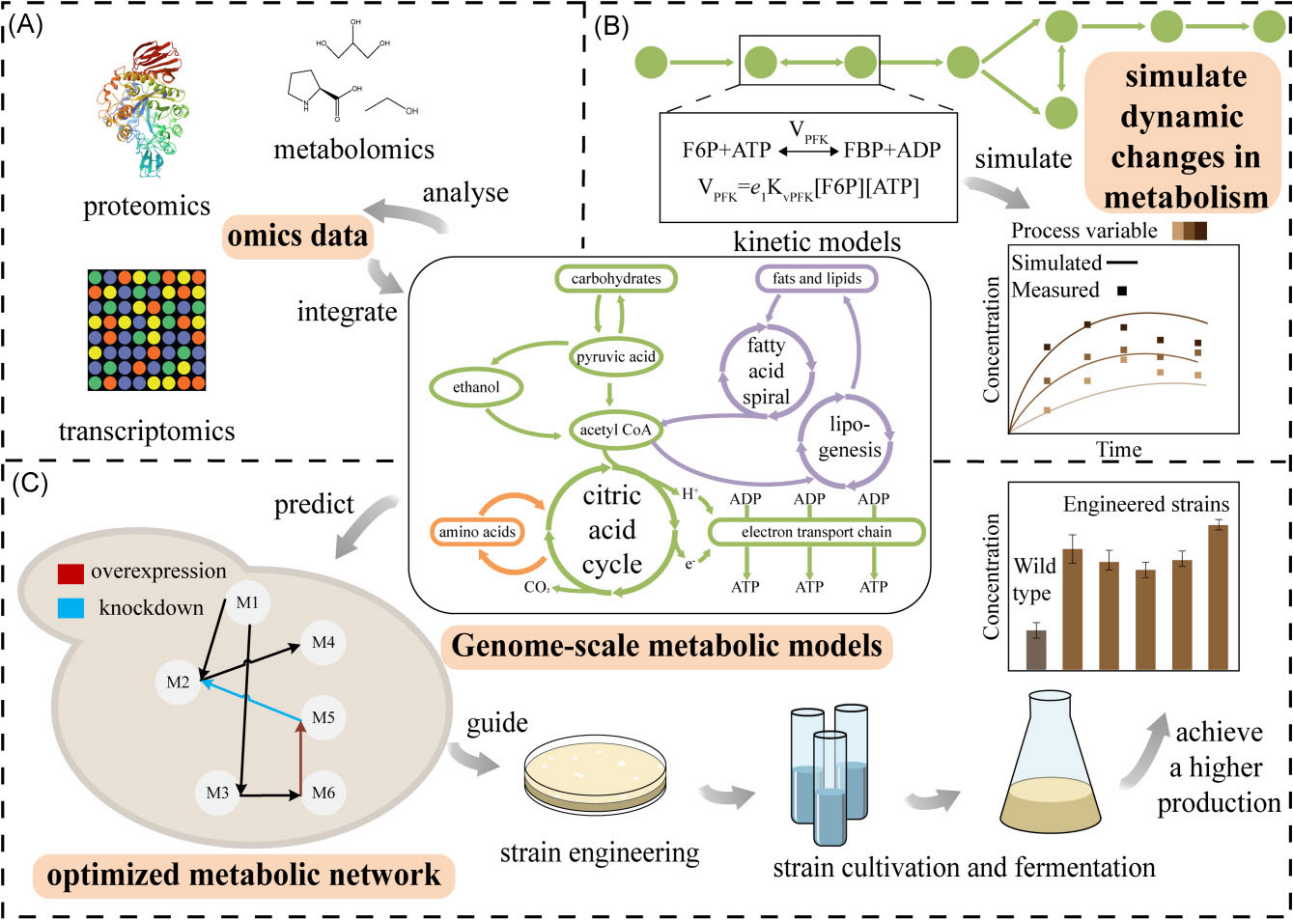
Applications of yeast GEMs. Integration and analysis of omics data, such as transcriptomics, proteomics, and metabolomics (A). Building on GEMs, kinetic models can predict the dynamics of cellular metabolism (B). Analysis of metabolic flux distributions and prediction of genetic targets for enhanced metabolite production (C).

GEMs could serve as powerful tools for integrating multidimensional omics datasets, which have been successfully applied in various areas, including systems biology and synthetic biology. In the following sections, we will introduce several key applications to demonstrate the unique value of these models in the above areas (Fig. 3).

## Integrative omics analysis

Yeast metabolic models have been widely used to integrate and interpret multiomics datasets. Meanwhile, the extra constraints from omics datasets could narrow solution spaces to enhance the prediction performances of yeast metabolic models (Chen et al. 2024a). In the aspect of integrative omics analysis, Culley et al. (2020) integrated Yeast7 with transcriptomics, successfully predicting yeast growth for 1143 single-gene knockout strains. Malina et al. (2021) displayed the mechanism underlying the emergence of the Crabtree effect by constraining ecGEMs with proteome abundance. Zhang et al. (2024) developed 163 single-cell GEMs with Yeast9 through the gene inactivity moderated by the metabolism and expression algorithm (Becker and Palsson 2008) from transcriptomics, revealing flux heterogeneity under osmotic stress. In a separate study, Hilsabeck and Rea (2024) leveraged Yeast8 to generate 812 viable single-gene knockout mutants, producing a total of 406 500 flux distributions. With deep learning models, the intricate relationship between critical reaction fluxes within the yeast metabolic network and replicative lifespan was elucidated. It revealed that each metabolic flux configuration corresponds to an inherent survival probability, suggesting that lifespan is an emergent property of these configurations. Intriguingly, despite the theoretically vast array of possible flux configurations (constrained by physicochemical limits), they found that these configurations converge into three distinct pseudometabolic states, each associated with significant shifts in survival probability. Moreover, to investigate the metabolic impacts of single-nucleotide polymorphism (SNP)–SNP interactions during yeast sporulation, Sasikumar et al. (2025) integrated SNP-specific GEMs with transcriptomic data, employing flux enrichment analysis to identify key metabolic reactions associated with sporulation, thereby elucidating SNP-driven metabolic flux alterations and their regulatory mechanisms. This progress illustrates that the integration and analysis of omics data with metabolic models could not only enhance the model prediction capabilities but also help to characterize yeast metabolism at a higher resolution.

## Elucidating the dynamic changes in yeast metabolism

GEMs have emerged as powerful tools in systems biology, enabling the simulation of yeast cell metabolic behavior under different growth environments. For instance, Henriques et al. (2021) recognized the limitations of steady-state assumptions and emphasized the importance of studying yeast metabolism during batch fermentation, a mode widely used in industrial production. To address this, they refined Yeast8 by integrating previously overlooked reactions and metabolites, developing a multiphase, multiobjective dynamic genome-scale model. This advanced model facilitates the analysis of dynamic shifts in both primary and secondary metabolism of yeast throughout batch cultivation, offering a more comprehensive view of metabolic behavior under industrially relevant conditions. Moimenta et al. (2025) integrated this enhanced Yeast8 model (Henriques et al. 2021) and a continuous model that simulates the physiological dynamics of yeast during wine fermentation (Moimenta et al. 2023), to create a sophisticated multiphase continuous model. This integrated framework enables the prediction of metabolic transitions across multiple growth phases in batch fermentation, including the lag, exponential growth, growth-non-growth transition, stationary, and decline phases, thus providing a novel lens to explore dynamic metabolic responses of yeasts along with different growth phases.

## Facilitate the rational design of cell factories

GEMs could facilitate the rational design and optimization of yeast metabolism via the simulation and prediction of metabolic behaviors. For instance, Li et al. (2022a) utilized the proteome-constrained pcSecYeast model, integrated with Flux Scanning based on Enforced Objective Function, to pinpoint overexpression targets for α-amylase secretion, achieving the experimental validation of 9 out of 14 predictions. Similarly, Sjöberg et al. (2024) validated the accuracy of ecGEMs in predicting metabolic shifts during yeast engineering for the co-production of 2,3-butanediol and glycerol, with model predictions closely consistent with experimental results. Moreover, Carruthers et al. (2025) applied kinetic modeling for eight reactions to optimize the mevalonate pathway, resulting in significant enhancements in prenol production. Additionally, Domenzain et al. (2025) introduced ecFactory, a platform incorporating enzyme kinetics into ecGEMs, to identify the gene targets for 103 high-value metabolites, thereby facilitating global strain optimization. Collectively, these findings emphasize the central role of advanced metabolic models in driving metabolic engineering for biotechnological applications.

Beyond pathway optimization, GEMs can also be used to assess the production potential of different chassis strains. Kim et al. (2025) constructed 1360 GEMs across six species, including *S. cerevisiae*, under varying aeration and carbon source conditions, evaluating their capacity to produce 235 bio-based chemicals through maximum theoretical and achievable yields. This comprehensive resource aids in selecting optimal hosts for bio-based production. By bridging predictive modeling with empirical validation, GEMs provide a robust framework for advancing yeast-based biomanufacturing.

## Conclusions and perspectives

Yeast GEMs have evolved gradually from early models like iFF708 to the comprehensive models like Yeast9, with enhanced scale, accuracy, and integration of biochemical data, including thermodynamics, and multiomics profiles. Besides, multiscale models, such as ecGEMs and whole-cell models, have improved the modeling of yeast metabolism under diverse conditions. These models facilitate the integration of omics data, elucidate the dynamic metabolic shifts, and provide guidance for metabolic engineering.

Despite these achievements, the accurate modeling of yeast metabolism at the basic metabolic network level faces several challenges. Firstly, classical yeast GEMs still face limitations such as incomplete representations of lipid metabolism, as observed in recent models such as Yeast9 (Hilsabeck and Rea 2024). This deficiency restricts the accurate simulation of critical cellular processes, including membrane dynamics and signaling. This issue might be alleviated by integrating comprehensive lipidomics data into these models, thereby enhancing the more accurate representation of lipid metabolism. Additionally, the functional ambiguity of numerous genes could lower the quality of yeast GEMs, requiring deeper investigation into gene functions through functional genomics approaches (Wang et al. 2024). Furthermore, oversimplified assumptions, such as constant biomass composition under diverse conditions, lead to inaccurate flux predictions (Sjöberg et al. 2024). Moreover, when building GEMs for nonmodel organisms, draft models generated by automated algorithms often exhibit limited accuracy, which necessitates manual curation and targeted experimental validation. This limitation poses a bottleneck to high-throughput generation of high-quality models. Besides, the application of most multiscale models faces challenges, including limited transferability to nonmodel organisms and a lack of user-friendly software tools, which hinders their widespread use in systematic strain development. These limitations will be the driving force for a new round of rapid development of yeast GEMs.

Recent advancements in computational tools and artificial intelligence have brought new promise to the enhancement and extension of yeast GEMs applications. For example, the toolboxes, like METAFlux (METAbolic Flux balance analysis) (Huang et al. 2023) and ftINIT (fast task-driven integrative network inference for tissues) (Gustafsson et al. 2023), could integrate single-cell transcriptomics and phenotypic data with GEMs to improve flux predictions, providing insights into metabolic diversity at the single-cell level. Additionally, artificial intelligence has accelerated the development of computational models (Lu et al. 2024). For instance, the automatically GEMs reconstruction method CarveFungi employs machine learning to assign proteins to distinct cellular compartments (Castillo et al. 2023). Furthermore, machine learning algorithms can address gaps in metabolic networks, exemplified by CHESHIRE (CHEbyshev Spectral HyperlInk pREdictor), a hypergraph learning-based algorithm to fill gaps in draft GEMs (Chen et al. 2023). For multiscale models, machine learning facilitates parameter estimation. The latest versions of ecGEMs tools, GECKO 3.0 and ECMpy 2.0, both enhance the coverage of *k*_cat_ by integrating DLKcat (Li et al. 2022b), a machine learning method for predicting enzyme kinetic parameters. Beyond DLKcat, recent advancements in *k*_cat_ prediction include methods such as TurNup (Kroll et al. 2023), UniKP (Yu et al. 2023), DeepEnzyme (Wang et al. 2024), CatPred (Boorla and Maranas 2025), and PreTKcat (Cai et al. 2025), which demonstrate ongoing efforts to optimize the estimation of enzyme kinetic parameters. Together, it is anticipated that the quality of yeast GEMs and the derived multiscale models will be further improved, thus playing an increasingly pivotal role in numerous yeast metabolism studies.
